# Draft genome sequences for *Enterococcus mundtii* isolates from soil in Minnesota

**DOI:** 10.1128/mra.00249-26

**Published:** 2026-06-15

**Authors:** Erna Karic, Amanda L. Haeberle, Julia L. E. Willett

**Affiliations:** 1 Department of Microbiology and Immunology, University of Minnesota Medical School 12269, Minneapolis, Minnesota, USA; University of Pittsburgh School of Medicine, Pittsburgh, Pennsylvania, USA

**Keywords:** *Entercoccus mundtii*, enterococci, soil bacteria, whole-genome sequencing

## Abstract

*Enterococcus mundtii* is a gram-positive bacterium found in the environment and mammalian gastrointestinal tracts and is an infrequent cause of infections in humans. We describe the isolation and sequencing of three closely related isolates of *E. mundtii* obtained from soil. These strains will be useful for work on understudied enterococci.

## ANNOUNCEMENT

*Enterococcus* species are gram-positive, facultative anaerobic bacteria frequently found as gastrointestinal tract commensals in mammals, birds, and insects, and these bacteria are prevalent in the environment (1–4). The best-studied enterococcal species, *Enterococcus faecalis* and *Enterococcus faecium*, cause infections in humans and can be highly resistant to antimicrobials (5). Other enterococci are relatively understudied, yet display interesting phenotypes such as pigmentation and motility (6, 7). This study presents the draft genome sequences of three closely related isolates of *Enterococcus mundtii*, which are pigmented and non-motile (8, 9). Soil samples were collected in collaboration with the MICB 4215 course at the University of Minnesota-Twin Cities during October 2023. Approximately 0.5 grams of soil were resuspended in 0.5 mL sterile water, and 0.1 mL was plated on both tryptic soy agar (TSA) and bile esculin azide (BEA) agar, a selective medium for enterococci. Plates were incubated for 24 h at 37°C. Three presumptive *Enterococcus* isolates (dark colonies on BEA plates) were obtained from a soil sample taken after a rainstorm from beneath a large mushroom near the University of Minnesota. These three isolates, designated 4215-23-A1 through C1, were chosen for further analysis due to their yellow pigmentation on TSA plates, which differs from other enterococci such as *E. faecalis*. All putative enterococci were restreaked, grown in tryptic soy broth (TSB), and stored at −80°C in TSB supplemented with 25% glycerol.

Samples from freezer stocks were grown statically in TSB for 24 h at 37°C. DNA was isolated using a Qiagen DNeasy Blood and Tissue Kit with a lysozyme pre-treatment as previously described for *E. faecalis* (10). Concentration of DNA was measured using Qubit, and DNA purity was assessed using a NanoDrop. Library preparation and whole-genome sequencing were performed at SeqCoast. Libraries were prepared with the Illumina DNA Prep tagmentation kit and IDT for Illumina Unique Dual Indexes. An Illumina NextSeq2000 (300 cycle flow cell, 2 × 150 bp paired-end reads) was used for sequencing. DRAGEN (v3.10.12) was used for demultiplexing, read analytics, and trimming. Read quality was inspected using FastQC (11), and reads were assembled in Unicycler v0.4.8 (12) and annotated using the NCBI Prokaryotic Genome Annotation Pipeline version 6.10 (13). Samples were identified as *E. mundtii* using the Bacterial and Viral Bioinformatics Resource Center (BV-BRC) Taxonomic Classification Service (14). This classification was consistent with colony phenotypes, specifically production of yellow pigment. Isolate information is provided in Table 1. No plasmids were identified in any isolate using PlasmidFinder-2.0 (15) with the default settings or by inspecting Bandage plots (16) in BV-BRC. All strains had 49 transfer RNAs and 2 16S ribosomal RNA genes. All samples had >99% completeness with 0.16%–0.26% contamination as evaluated by CheckM (v1.2.4) (17).

**TABLE 1:** Genome information and GenBank assembly statistics for *Enterococcus mundtii* isolates from soil

	*Enterococcus mundtii* isolates		
	4215-23-A1	4215-23-B1	4215-23-C1
Genome size (bp)	3,122,934	3,040,745	3,051,831
Contigs	67	53	59
Genome coverage	99×	102×	135×
N50 (bp)	237,970	238,061	238,097
Genes	2,959	2,870	2,892
Protein-coding genes	2,859	2,770	2,795
GC content (%)	38	38.5	38.5
Sequence Read Archive (SRA) accession	SRR35794999	SRR35794998	SRR35794997
BioSample accession number	SAMN52748871	SAMN52748872	SAMN52748873
GenBank assembly number	GCA_054482215.1	GCA_054482255.1	GCA_054482235.1
ANI match to type strain DSM 4838 (%)	96.5	96.5	96.5

## Data Availability

Raw reads from all *E. mundtii* isolates were deposited with BioProject number PRJNA1345430. Isolate accession numbers in Sequence Read Archive (SRA) are SRR35794999 (A1), SRR35794998 (B1), and SRR35794997 (C1). The GenBank assembly of type strain *Enterococcus mundtii* DSM 4838 is GCA_007990125.1.
